# The Connectomic Glutamate Framework for Depression: Bridging Molecular Plasticity and Network Reorganization

**DOI:** 10.3390/brainsci16010018

**Published:** 2025-12-24

**Authors:** Pietro Carmellini, Mario Pinzi, Maria Beatrice Rescalli, Alessandro Cuomo

**Affiliations:** Department of Molecular and Developmental Medicine, Division of Psychiatry, University of Siena School of Medicine, 53100 Siena, Italy

**Keywords:** connectome, glutamatergic signaling, major depressive disorder, network dysfunction, synaptic plasticity

## Abstract

Major depressive disorder (MDD) is increasingly recognized as a disorder of impaired neuroplasticity and large-scale network dysfunction rather than a simple monoaminergic deficit. Converging evidence indicates that chronic stress and depression erode synaptic connectivity, reduce glial support, and destabilize functional interactions among the default mode, salience, and executive networks. Conventional antidepressants indirectly restore circuit function over weeks, but the advent of rapid-acting glutamatergic agents has opened a new path for targeting these abnormalities directly. In this narrative review, we synthesize molecular, cellular, and connectomic findings to outline a conceptual Connectomic Glutamate Framework of Depression. We first examine how NMDAR blockade and subsequent AMPAR facilitation activate mTORC1 and BDNF signaling, driving synaptogenesis and dendritic spine formation. We then highlight the role of astrocytes and microglia in shaping the “quad-partite synapse” and sustaining network integrity. Neuroimaging studies demonstrate that glutamatergic modulators remodel dysfunctional networks: dampening DMN hyperconnectivity, enhancing fronto-limbic coupling, and normalizing salience-driven switching. Integrating these domains, we propose a hypothesis-generating, two-phase model in which glutamatergic agents destabilize maladaptive attractor states and then reintegrate circuits through structural remodeling. This framework bridges molecules, cells, and networks, offering mechanistic insight into the rapid efficacy of glutamatergic antidepressants and highlighting priorities for clinical translation.

## 1. Introduction

Major depressive disorder (MDD) is increasingly recognized as a disorder of impaired neuroplasticity and network dysfunction rather than a simple monoaminergic imbalance [1,2]. While traditional monoamine-based theories focusing on deficits in serotonin, norepinephrine, or dopamine have provided an important framework, they fail to account for the substantial heterogeneity of depressive phenotypes and the characteristic delay in the therapeutic effects of conventional antidepressants [3,4].

In contrast, converging lines of evidence implicate widespread structural and functional brain changes in MDD, including loss of synapses and glial elements as well as maladaptive activity within large-scale networks [5,6,7]. Such abnormalities have been consistently identified across cellular, glial, and connectomic domains, as evidenced by convergent findings from postmortem investigations, positron emission tomography (PET) imaging, and functional magnetic resonance imaging (fMRI) studies. Together, they point to depression as a disorder of impaired plasticity and network integration, undermining the brain’s ability to sustain adaptive cross-circuit communication [8,9]. Within this neurocircuitry framework, treatments capable of enhancing synaptic plasticity and recalibrating large-scale networks can be promising.

Conventional antidepressants are now recognized to exert their effects, at least in part, through the gradual upregulation of neurotrophic factors such as brain-derived neurotrophic factor (BDNF), thereby promoting synaptogenesis and even hippocampal neurogenesis, ultimately restoring circuit integrity over weeks [10]. However, a major paradigm shift occurred with the discovery that antagonism of the N-methyl-D-aspartate (NMDA) receptor could induce rapid antidepressant effects. The landmark placebo-controlled trial of ketamine by Berman et al. (2000) demonstrated, for the first time in humans, that depressive symptoms can be alleviated within hours of a single infusion [11]. Subsequent trials confirmed these effects in treatment-resistant depression (TRD), firmly establishing ketamine as the prototype of rapid-acting glutamatergic antidepressants [12,13].

These findings opened a new path for understanding depression as a disorder of maladaptive connectomic states that can be rapidly “reset” through glutamate-driven plasticity. By engaging α-amino-3-hydroxy-5-methyl-4-isoxazolepropionic acid (AMPA) receptor throughput, activating mammalian target of rapamycin complex 1 (mTORC1) signaling, and engaging BDNF-tropomyosin receptor kinase B (TrkB) cascades, glutamatergic agents promote synaptogenesis and circuit reorganization, which ultimately scale up to network-level normalization [14,15,16]. Neuroimaging studies confirm that such interventions can modulate dysfunctional connectivity, providing translational evidence that molecular and synaptic changes drive systems-level reorganization [17,18].

While most reviews have focused either on molecular mechanisms or on network dysfunction, here we propose an integrative framework that explicitly links molecular and cellular plasticity with connectomic reorganization.

In this narrative review, we therefore aim to: (i) Summarize key molecular and cellular mechanisms implicated in rapid-acting glutamatergic antidepressants, including NMDAR blockade, AMPA facilitation, mTORC1 activation, and BDNF–TrkB signaling; (ii) Highlight the role of astrocytes and microglia in shaping synaptic and network plasticity, within the concept of the “quad-partite synapse”; (iii) Synthesize neuroimaging and connectomic evidence on large-scale network alterations in MDD and their modulation by glutamatergic agents and (iv) Propose the Connectomic Glutamate Framework of Depression as a conceptual, integrative model that links molecular, cellular, glial, and network domains, outlining testable hypotheses and key gaps for future empirical validation.

Our goal is not to provide a systematic or exhaustive synthesis of all available data, but rather to offer a mechanistically oriented, multi-level perspective that may help organize a heterogeneous literature and guide future experimental and clinical research [5,19].

## 2. Materials and Methods

This article is a narrative review, aimed at synthesizing mechanistic, cellular, glial, and connectomic evidence relevant to glutamatergic antidepressants. As such, it does not adhere to PRISMA guidelines and does not employ a systematic or reproducible search strategy, nor does it include predefined eligibility criteria, structured study selection, or formal quality appraisal. The purpose of the review is conceptual rather than exhaustive, focusing on the integration of key findings to formulate a hypothesis-generating framework. To inform the narrative synthesis, we conducted a broad, non-systematic search of the literature using PubMed, Web of Science, and Google Scholar (up to September 2025). Search terms included pharmacological agents (“ketamine”, “esketamine”, “dextromethorphan-bupropion”), molecular and cellular pathways (“NMDA receptor blockade”, “AMPA potentiation”, “mTOR signaling”, “BDNF-TrkB”, “synaptogenesis”, “astrocytes”, “microglia”), and network or connectomic concepts (“default mode network”, “central executive network”, “salience network”, “fronto-limbic connectivity”, “network remodeling”). Additional keywords addressed translational aspects (“EEG biomarkers”, “PET synaptic density”, “treatment durability”, “precision psychiatry”). Because the review is narrative in nature, no structured screening protocol or reproducible Boolean strategy was implemented. Searches were exploratory and iterative, designed to identify influential publications across different domains of evidence rather than to retrieve all available studies. Conceptual criteria guided study selection, including: Mechanistic relevance (evidence linking glutamatergic modulation to synaptic plasticity, glial function, or molecular pathways such as AMPA, mTORC1, or BDNF–TrkB), translational importance, representative or landmark contributions (e.g., first demonstration of rapid ketamine effects, first connectomic evidence, pivotal enantiomer/metabolite studies) and complementarity across levels of analysis (molecular, cellular, glial, systems-level).

From this broad search, we identified 21 studies that were considered “pivotal” based on their high mechanistic relevance, novelty, or translational impact. These articles were not selected through a systematic or quantitative process but rather chosen for their conceptual importance in shaping the integrative framework presented in this review. They were therefore organized into two domains:

1. glutamatergic mechanisms and rapid-acting antidepressants, and
2. network-level findings and connectomic remodeling.

Given the heterogeneity of study designs and the narrative purpose of the review, no meta-analysis, risk-of-bias assessment, or formal grading of evidence was conducted. The review aims to synthesize convergent themes while acknowledging methodological variability across the literature.

## 3. Depression as a Network Dysfunction: From Monoamines to Circuits

Monoaminergic antidepressants acutely elevate neurotransmitter levels, yet their clinical benefits emerge only after weeks of treatment, and up to one-third of patients remain nonresponsive [4,19]. These limitations highlight the need for circuit-level explanations that better capture the core features of MDD.

Neuroimaging consistently shows that MDD involves disturbances in large-scale brain networks [2,20,21,22,23]. The default mode network (DMN), encompassing the medial prefrontal cortex (mPFC) and posterior cingulate cortex (PCC), is hyperconnected and hyperactive in depression, driving pathological rumination and difficulty disengaging from negative self-focus [2,24,25]. In contrast, the central executive network (CEN), anchored in the dorsolateral prefrontal cortex (dlPFC), is under-engaged, while the salience network (SN), centered on the dorsal anterior cingulate (dACC) and anterior insula, shows impaired switching between DMN and CEN states [26]. This triple-network dysfunction, DMN hyperconnectivity, CEN hypoactivity, and SN dysregulation—closely tracks symptom severity, with greater DMN abnormalities predicting worse rumination and cognitive rigidity [20,21,23,24].

Structural and cellular pathology reinforce these functional deficits. Chronic stress and recurrent depression lead to neuronal atrophy, dendritic retraction, and spine loss in the prefrontal cortex and hippocampus [8]. Postmortem studies reveal reduced astrocytic and oligodendrocytic density in fronto-limbic regions such as the sgACC and dlPFC, implicating glial pathology [27]. Dysregulation of astrocytic glutamate transporters (e.g., GLT-1) and glutamine synthetase further suggests impaired glutamate clearance and synaptic support [28]. Transcriptomic studies corroborate these findings, reporting decreased expression of synaptic genes and markers of synaptic density [6].

In vivo imaging supports this cellular pathology; PET studies with SV2A ligands show reduced synaptic density in the hippocampus and PFC, correlating with both altered connectivity and symptom severity [29]. Aberrant DMN-sgACC coupling has also been directly linked to affective disturbances [30].

Taken together, these data suggest that the loss of synaptic integrity and glial support may degrade the neurobiological infrastructure needed for flexible network coordination. This breakdown leads to a dominance of internally focused, rigid brain states—anchored in the DMN—and a diminished capacity for external engagement and adaptive control via the CEN and SN. These network-level abnormalities do not arise in isolation but are grounded in underlying synaptic and cellular dysfunction, pointing to the need for models that connect molecular pathology to systems-level changes.

In this context, converging transcriptomic evidence indicates that oligodendrocyte precursor cells (OPCs) represent one of the most prominently dysregulated glial populations in MDD. Single-nucleus analyses of the prefrontal cortex have identified immature OPCs as disproportionately affected, with gene expression changes consistent with impaired differentiation, increased apoptotic vulnerability, and disrupted myelin-related programs. These findings implicate altered OPC maturation and oligodendrocyte plasticity as potential contributors to white-matter and network-level dysfunction in depression [31,32].

Despite mounting evidence implicating glutamatergic dysregulation in MDD, current findings remain heterogeneous and are often constrained by small sample sizes, methodological inconsistencies, and a lack of longitudinal data.

## 4. Molecular and Cellular Mechanisms

To maintain clarity, mechanistic descriptions in this section focus on cellular and molecular events, whereas clinical implications are addressed separately in Section 6. The two domains are conceptually linked but should not be conflated, as molecular findings do not directly translate to clinical outcomes.

### 4.1. NMDA Receptor Blockade and AMPA Throughput

A defining feature of rapid-acting glutamatergic antidepressants is their ability to trigger a cascade of plasticity-related events within hours of administration. At sub-anesthetic doses, ketamine acts as a non-competitive NMDA receptor (NMDAR) antagonist, although its precise cellular targets and sequence of events are still debated. Preclinical models suggest preferential blockade of NMDARs on cortical GABAergic interneurons, reducing inhibitory tone on pyramidal neurons and thereby eliciting a transient glutamate burst in the medial prefrontal cortex (mPFC) and hippocampus [33,34,35,36]. However, interneuron–pyramidal dynamics may vary across cortical layers, species, and experimental conditions, and human studies cannot directly confirm these cellular mechanisms. Some models also propose contributions from direct actions on pyramidal neurons or other cell types, highlighting the complexity of translating rodent circuitry to humans.

The resulting glutamate surge enhances AMPA receptor throughput, which is indispensable for antidepressant efficacy; AMPA antagonists abolish ketamine-induced synaptic protein upregulation and behavioral responses [37,38]. Thus, the benefit of ketamine stems not from NMDA antagonism itself, but from secondary AMPA engagement, although the degree to which AMPA potentiation alone explains ketamine’s clinical effects remains an open question, especially in humans. In the connectomic framework, AMPA-driven excitatory plasticity provides the synaptic “push” needed to destabilize rigid depressive network states, initiating large-scale reorganization [39].

### 4.2. mTORC1 Signaling and Synaptogenesis

AMPA receptor activation recruits the mammalian target of rapamycin complex 1 (mTORC1), a key regulator of dendritic protein synthesis and synapse formation. Within 1–2 h of ketamine administration, mTOR phosphorylation in the PFC increases, accompanied by upregulation of PSD-95, GluA1, and synapsin I, and a surge in dendritic spine density [14].

Nevertheless, mTORC1 activation has not been uniformly observed across all preclinical studies, and human evidence remains indirect [40]. In addition, the relative contribution of mTORC1 versus parallel pathways (e.g., ERK, GSK3 inhibition) is still under investigation. The mTOR inhibitor rapamycin completely eliminated both synaptic and behavioral effects of ketamine which proved that mTORC1 functions as a critical component in its mechanism, but the generalizability of these findings to humans is limited by dose, timing, and species differences. The mTOR pathway is not unique to ketamine but appears to be a shared mediator across glutamatergic antidepressants. The two medications activate identical plasticity-related pathways but at a reduced speed when used together instead of individually. The study confirms that mTORC1 activation functions as a common biological pathway which leads to fast antidepressant effects. The intracellular “translator” mTORC1 functions as a systems neuroscience component which transforms AMPA-induced synaptic excitation into enduring structural changes [5,41].

The transient glutamate burst leads to enduring network reconfiguration through mTOR signaling which enables de novo protein synthesis and spine formation.

### 4.3. BDNF-TrkB Signaling

In parallel with mTOR activation, brain-derived neurotrophic factor (BDNF) is indispensable for the antidepressant actions of ketamine. Under normal conditions eukaryotic elongation factor 2 kinase (eEF2K) blocks the translation of BDNF.

Ketamine works quickly to block eEF2K which removes the inhibitory effect and enables a rapid increase in BDNF protein production [42]. However, the magnitude and timing of BDNF changes following ketamine differ across species, strains, brain regions, and experimental paradigms. Human data are also heterogeneous, with some studies showing increases in circulating BDNF and others finding no clear association. BDNF activates TrkB receptors to initiate downstream signaling pathways (ERK, CREB), supporting dendritic spine growth and synaptic enhancement [43]. Liu et al. showed that TrkB signaling pathways play a vital role in ketamine behavioral effects because they found that blocking these pathways through genetic or pharmacological methods eliminated all therapeutic effects of ketamine. The Val66Met BDNF polymorphism which hinders BDNF release during activity leads to reduced antidepressant effects of ketamine in people who carry this genetic variation [15]. Still, the exact contribution of peripheral versus central BDNF changes, and the extent to which rodent TrkB dynamics map onto human depression, remain uncertain.

The research shows BDNF–TrkB signaling functions as a standalone mechanism which produces fast antidepressant effects. BDNF functions as a molecular messenger that enables synaptic plasticity to establish functional neural circuits through its role as a plasticity enabler, but its role is best viewed as contributory rather than singular or deterministic. The connectomic framework depends on BDNF release to convert glutamate destabilization into adaptive network reorganization instead of random neural activity.

### 4.4. Spine Formation and Structural Plasticity

Stress-induced depression models consistently show dendritic retraction and spine loss in the mPFC and hippocampus, correlating with cognitive and affective impairments [8]. These structural changes mirror postmortem findings in MDD patients, where synapse loss and reduced glial support are prominent. Ketamine has been shown to reverse stress-induced structural deficits. Within 24 h, treated animals exhibit increased dendritic complexity and spine density, effectively restoring microcircuit integrity. Moda-Sava et al. (2019) demonstrated that these newly formed spines are functionally indispensable: selective ablation of ketamine-induced spines using a chemogenetic tool eliminated the sustained antidepressant effect, even though acute behavioral improvement persisted briefly [16]. Thus, new spine formation is necessary for durability, providing a mechanistic explanation for why ketamine can maintain clinical benefits days after the drug is cleared from the body.

From the network perspective, spine formation equates to microcircuit reconnection, supplying the wiring needed to rebalance large-scale networks. In this sense, spines act as the “building blocks” of the connectome’s recovery.

### 4.5. Enantiomers and Metabolites

The story of mechanism of ketamine has been further refined by investigations into its enantiomers and metabolites. Preclinical work suggests that R-ketamine may produce more sustained antidepressant effects than S-ketamine (esketamine), with a more favorable side effect profile despite lower NMDAR affinity [44]. Nevertheless, human evidence directly comparing enantiomers is limited, and results remain preliminary. Mechanistically, R-ketamine seems to preferentially engage synaptogenic pathways and sustain BDNF elevation, raising the possibility of its future clinical development as a longer-lasting alternative. Equally important are metabolites of ketamine, particularly (2R,6R)-hydroxynorketamine (HNK). Zanos et al. (2016) demonstrated that HNK exerts antidepressant-like effects independently of NMDAR antagonism, instead acting via direct AMPA receptor potentiation [45]. HNK produces robust behavioral improvements in rodents without dissociative side effects, suggesting that downstream AMPA facilitation may suffice to trigger the antidepressant cascade. However, the translational relevance of HNK is debated: human studies have not consistently demonstrated robust antidepressant effects attributable to HNK levels, and some researchers argue that rodent HNK mechanisms may not generalize to humans. The clinical significance of HNK therefore remains uncertain and requires further investigation.

Together, these findings support the idea that the antidepressant core lies not in NMDA blockade itself but in AMPA-mediated plasticity, with different compounds or metabolites offering variations in potency, safety, and duration.

### 4.6. Convergence with Dextromethorphan-Bupropion

While most mechanistic insights derive from ketamine, emerging evidence indicates that dextromethorphan (DXM), in combination with bupropion, engages convergent pathways. DXM provides NMDA antagonism and sigma-1 receptor agonism, both of which promote BDNF release and plasticity. Nevertheless, DXM’s pharmacodynamic complexity, rapid metabolism, and species differences make its mechanistic profile more difficult to map onto the canonical ketamine cascade. The addition of bupropion prolongs DXM exposure and contributes to its own noradrenergic-dopaminergic activity. Clinical findings show that patients treated with DXM-bupropion can experience symptom improvement within one week, consistent with a glutamate-driven mechanism that overlaps with ketamine but unfolds more gradually [46,47], though the exact overlap with ketamine mechanisms remains to be clarified. This convergence highlights that glutamatergic antidepressant, despite pharmacological heterogeneity, likely share a final pathway of BDNF-mTOR-AMPA-driven synaptogenesis.

**Figure 1:**
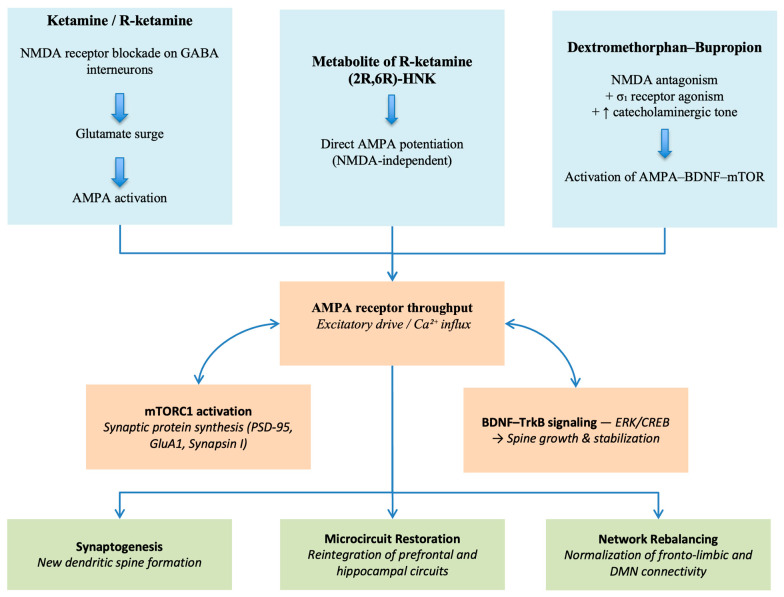
Molecular and cellular cascade underlying rapid glutamatergic antidepressant action. NMDAR blockade on GABAergic interneurons induces a glutamate surge that enhances AMPAR throughput, activating mTORC1 and BDNF–TrkB signaling. These pathways promote synaptogenesis, dendritic spine formation, and glial-mediated stabilization, enabling restoration of microcircuit integrity in prefrontal and hippocampal regions.

Importantly, DXM-bupropion demonstrates that these mechanisms can be harnessed in an orally administered agent, expanding accessibility and underscoring the therapeutic versatility of the glutamatergic approach (Figure 1).

## 5. Glial Modulation of Plasticity

### 5.1. Astrocytes and Synaptic Regulation

While much of the attention on rapid-acting antidepressants has focused on neuronal synapses, an equally important dimension involves the contribution of glial cells to glutamatergic signaling and synaptic plasticity. Evidence from both preclinical and postmortem studies consistently indicates that astrocytic and microglial dysfunction plays a pivotal role in the pathophysiology of MDD, although most available findings are correlational and do not establish direct causality [7,48,49].

Astrocytes are essential regulators of synaptic homeostasis. They maintain extracellular glutamate levels through high-affinity transporters such as GLT-1 (EAAT2) and GLAST (EAAT1), preventing excitotoxic accumulation while ensuring precise temporal and spatial control of synaptic transmission [50]. In addition, astrocytes release gliotransmitters, including ATP and D-serine, and secrete trophic factors such as BDNF and VEGF that directly influence synaptic plasticity [51].

Postmortem studies of depressed patients have revealed reduced astrocytic density in cortical and limbic regions, particularly in the subgenual anterior cingulate cortex (sgACC) and dorsolateral prefrontal cortex (dlPFC), regions also implicated in network-level dysfunction [27].

Transcriptomic analyses further indicate downregulation of genes associated with astrocytic glutamate transport and metabolism, suggesting impaired glutamate clearance and diminished metabolic support for neurons [28]. However, these observations do not clarify whether astrocytic abnormalities contribute to depressive symptoms, result from chronic stress or illness progression, or reflect compensatory processes. The causal directionality remains largely unresolved. The causal directionality remains largely unresolved.

### 5.2. Microglia and the Quad-Partite Synapse

Microglia, the resident immune cells of the brain, also exert profound influence on synaptic connectivity. Beyond their immune functions, microglia actively participate in synaptic pruning, surveillance, and remodeling, processes that are critical for maintaining circuit homeostasis [52]. In depression, microglial activation often shifts toward a pro-inflammatory, “M1-like” phenotype, releasing cytokines such as IL-1β and TNF-α that impair synaptic plasticity and reduce BDNF availability [53]. Yet, evidence in humans remains indirect, and it is unclear whether microglial alterations represent a driver, marker, or downstream consequence of depressive pathology. Conversely, microglia in an anti-inflammatory, “M2-like” state promote neuroprotection and facilitate synaptogenesis through secretion of trophic factors. Preclinical work indicates that ketamine rapidly modulates microglial activity, attenuating pro-inflammatory signaling while enhancing microglial-derived BDNF release [54]. Still, these results derive almost entirely from animal studies, and their translational relevance requires caution. These effects may contribute to the drug’s ability to normalize fronto-limbic connectivity and reduce depressive behaviors.

Together, astrocytes and microglia interact with neurons to form the so-called “quad-partite synapse”, where pre- and postsynaptic neuronal compartments are dynamically regulated by glial partners [55,56,57]. This framework is conceptually informative, but empirical support in humans is limited, and current data do not allow definitive causal inferences about glial contributions to MDD or glutamatergic antidepressant effects. Nevertheless, this framework underscores that synaptic plasticity is not solely a neuronal phenomenon but emerges from the integrated contributions of glial and neuronal elements. By influencing glutamate clearance, neurotrophic support, and synaptic pruning, glial cells provide the permissive environment in which glutamatergic antidepressants can exert their full efficacy. Importantly, clinical neuroimaging studies now support a glial contribution to network dysfunction. PET imaging with translocator protein (TSPO) ligands suggests abnormal microglial activation in depressed patients, particularly in the anterior cingulate and prefrontal regions [58], therefore provides correlational evidence of glial dysregulation rather than direct confirmation of causal microglial involvement. Yet TSPO PET suffers from several important limitations: TSPO expression is not specific to microglia and is also present in astrocytes, endothelial cells, and perivascular macrophages.

Similarly, reductions in SV2A binding indicate decreased synaptic density, but cannot distinguish between neuronal and glial contributions to synaptic loss [29]. In light of these limitations, the involvement of glial cells in rapid-acting antidepressant effects should be interpreted cautiously. While glutamatergic modulators may influence glial physiology, current evidence supports an associative rather than definitive causal relationship, particularly in humans. Glial contributions are therefore best viewed as potential modulators or facilitators of synaptic and network plasticity, rather than primary drivers of antidepressant response.

The concept of the quad-partite synapse underscores the importance of glia in maintaining synaptic homeostasis, but its role within MDD and ketamine response remains to be empirically validated through longitudinal, multimodal, and human-specific approaches. Overall, glial contributions should be viewed as modulatory rather than determinative; current evidence does not support a causal, glia-driven mechanism of antidepressant response, and interpretations must remain provisional.

## 6. Clinical Evidence for Rapid-Acting Glutamatergic Antidepressants

### 6.1. Ketamine: From Discovery to Clinical Application

Beyond the mechanistic insights discussed above, clinical development of ketamine has provided the most compelling evidence for rapid-acting glutamatergic antidepressants. As the prototype of this class, ketamine has been extensively investigated in randomized controlled trials, beginning with Berman et al. (2000) [11].

Preclinical studies dating back to the 1990s suggested that excessive NMDA receptor activity might contribute to depressive-like states and that pharmacological NMDA receptor antagonism could exert antidepressant properties [59]. These observations culminated in clinical investigations of ketamine, a noncompetitive NMDA receptor antagonist with a long history of use as an anesthetic.

In 2000, Berman and colleagues conducted the first placebo-controlled clinical trial demonstrating that a single low-dose ketamine infusion (0.5 mg/kg, IV) produced a rapid antidepressant effect within hours in patients with MDD [11]. This proof-of-concept finding was revolutionary, showing for the first time in humans that depressive symptoms could be alleviated almost immediately by a pharmacological agent, in stark contrast to the delayed action of monoaminergic antidepressants. Subsequent randomized controlled trials confirmed and extended these results. In 2006, Zarate et al. demonstrated in TRD that ketamine produced robust antidepressant effects within 24 h, with approximately 70% of patients meeting response criteria compared with fewer than 30% in the placebo group [12].

Notably, while the acute effects of a single ketamine infusion typically waned after 5–7 days, a substantial subset of patients maintained clinical response for a week or more. Meta-analyses have since confirmed rapid efficacy of ketamine in TRD, with response rates of 50–70% at 24 h, though relapse is common without repeated dosing [60]. These pioneering findings established ketamine as the prototype of a new class of rapid-acting antidepressants.

### 6.2. Esketamine: Translating Mechanism into Practice

The success of ketamine spurred the development of related agents, most notably esketamine, the S-enantiomer of ketamine. Esketamine has approximately fourfold greater affinity for the NMDA receptor than R-ketamine [44]. Based on Phase III trials, intranasal esketamine was approved by the U.S. Food and Drug Administration (FDA) in 2019 for TRD and later for depressive episodes with acute suicidal ideation or behavior [61]. In pivotal studies, adjunctive intranasal esketamine plus an oral antidepressant produced greater reductions in depression severity and higher remission rates than antidepressant plus placebo [62].

Moreover, the SUSTAIN-1 trial showed that maintenance treatment with esketamine delayed relapse compared with discontinuation, thereby demonstrating its utility not only for acute symptom relief but also for sustaining remission [61]. Like racemic ketamine, esketamine engages NMDA receptor blockade, AMPA throughput, and downstream mTOR-BDNF cascades, providing a molecular basis for its network-level effects. However, esketamine administration is restricted to certified clinics due to risks of transient dissociation, perceptual disturbances, and blood pressure elevations [62,63].

### 6.3. Dextromethorphan-Bupropion and Emerging Oral Agents

Another major advance has been the development of dextromethorphan-bupropion, the first oral, rapid-acting antidepressant approved by the FDA in 2022 [47]. Dextromethorphan (DXM), an antitussive agent, acts as an uncompetitive NMDA receptor antagonist and sigma-1 receptor agonist. However, rapid metabolism of DXM via CYP2D6 to dextrorphan limits its standalone efficacy. Co-formulation with bupropion, a norepinephrine-dopamine reuptake inhibitor and potent CYP2D6 inhibitor, prolongs DXM exposure while contributing additive antidepressant effects [64]. In the pivotal GEMINI trial, dextromethorphan-bupropion produced significantly greater improvements in depression severity than placebo, with benefits evident within the first week of treatment [47].

Compared with ketamine and esketamine, DXM-bupropion offers advantages in terms of oral administration, accessibility, and tolerability, though caution remains warranted regarding potential for misuse at supratherapeutic doses [47].

### 6.4. Novel and Experimental Compounds

Beyond approved therapies, numerous glutamate-based agents have been investigated. Rapastinel (GLYX-13), a glycine-site partial agonist of the NMDA receptor, initially showed promise as a non-dissociative intravenous agent with antidepressant activity, but failed to meet endpoints in Phase III trials [65]. Lanicemine (AZD6765), a low-trapping NMDA receptor antagonist, likewise did not significantly outperform placebo in pivotal studies despite transient clinical benefits [66]. Other compounds under investigation include AMPA receptor positive allosteric modulators (“ampakines”), which aim to enhance excitatory throughput and synaptic potentiation, and metabotropic glutamate receptor (mGlu) modulators, such as mGlu2/3 negative allosteric modulators, which can increase presynaptic glutamate release [67]. Although none have yet reached clinical application, these efforts highlight the broad therapeutic potential of targeting the glutamatergic system.

In summary, ketamine, intranasal esketamine, and oral dextromethorphan-bupropion represent a transformative therapeutic paradigm for depression. These drugs have fundamentally shifted the field by demonstrating that directly engaging glutamatergic signaling and synaptic plasticity can rapidly recalibrate dysfunctional networks in depression. Future development aims to refine these approaches by preserving rapid efficacy while minimizing adverse effects.

## 7. Network Remodeling and Connectomic Evidence

Glutamatergic modulators relieve depression not only by restoring local synaptic plasticity but also by reprogramming large-scale brain networks. Neuroimaging in humans and animal models consistently shows that ketamine and related agents rapidly reshape connectivity, effectively “resetting” the depressed connectome. The most robust changes involve the default mode network (DMN), fronto-limbic circuits, and the salience-executive system.

Recent large-scale and longitudinal neuroimaging studies indicate substantial interindividual variability in default mode network (DMN) and fronto-limbic alterations in major depressive disorder [20,68]. Rather than uniform DMN hyperconnectivity or fronto-limbic dysregulation, distinct connectivity subtypes have been identified, with both hyper- and hypoconnectivity patterns depending on symptom dimensions, illness stage, and treatment history [69]. Emerging evidence further suggests that glutamatergic interventions induce heterogeneous network reconfiguration trajectories, with baseline connectomic states modulating adaptive versus maladaptive remodeling [70].

### 7.1. Default Mode Network Normalization

A consistent finding is that ketamine reduces abnormally high DMN connectivity, a hallmark of MDD. Within 24 h of a single infusion, mPFC-DMN coupling decreases, correlating with clinical improvement [5,13]. Patients with the greatest baseline DMN overactivity show the strongest connectivity reductions and clinical benefit [18]. Thus, ketamine appears to “quiet” the DMN, alleviating rumination and negative self-focus.

Task-based studies confirm this effect, demonstrating ketamine-induced anterior DMN deactivation associated with fewer maladaptive self-referential thoughts [24]. Moreover, ketamine functionally decouples the habenula, normally hyperactive in depression and linked to anhedonia, from the DMN, effectively disconnecting a “punishment” signal that drives self-reproach [71]. This mechanism may underlie rapid mood-lifting and anti-anhedonic actions of ketamine.

### 7.2. Fronto-Limbic Reconnection

Alongside DMN normalization, ketamine strengthens connectivity between prefrontal regions and limbic structures central to emotion regulation. Within hours, dorsomedial and dorsolateral PFC show increased coupling with the amygdala and hippocampus [18,72], reflecting restored top-down control. Stronger PFC–amygdala connectivity correlates with clinical response and reduced negative affect [72]; in depression with PTSD, greater connectivity gains predict greater symptom relief [73].

Limbic hyperreactivity is also dampened; amygdala responses to negative emotional stimuli normalize within a day, and the extent of this reduction parallels mood improvement [74]. Collectively, these changes indicate that ketamine re-establishes balance between cognitive control and emotional processing.

### 7.3. Salience Network and Triple-Network Rebalancing

The salience network (SN), anchored in the dorsal anterior cingulate and anterior insula, also reorganizes rapidly after glutamatergic modulation. Ketamine recalibrates SN connectivity, restoring its role in shifting between internal and external modes. Abnormal SN coupling in MDD, excess DMN linkage, or weak executive integration normalizes after treatment, allowing more effective executive engagement and DMN suppression [17,18,24,61]. These adjustments align with the resolution of the triple-network dysfunction (see Section 8.1).

In addition, ketamine induces transient cortical changes, heightened excitability, gamma oscillations, and breakdown of rigid network segregation, interpreted as a destabilization phase linked to dissociation [17,75,76,77]. As drug effects subside, connectivity reintegrates along more adaptive patterns, with reduced DMN dominance and enhanced frontal activity [73]. Crucially, these effects depend on the glutamate surge: pretreatment with lamotrigine, which suppresses glutamate release, abolishes ketamine-induced network remodeling and its sustained antidepressant effects [78].

SN reconnection with DMN and CEN aligns with resolution of triple-network dysfunction; mechanistic interpretation is developed in Section 8.

## 8. The Connectomic Glutamate Framework for Depression: An Integrative Model

While earlier sections detail molecular, glial, and network-level findings, this section synthesizes those domains without reiterating mechanistic descriptions. Instead, the focus is on articulating how these processes may relate to one another within a conceptual scaffold.

### 8.1. Molecular-to-Network Cascade

The evidence reviewed across molecular, cellular, glial, and network domains converges on an integrated perspective, which we propose as the Connectomic Glutamate Framework of Depression. This framework is intended as a conceptual synthesis and heuristic rather than a definitive mechanistic model. It draws on convergent findings from preclinical and clinical studies, while acknowledging that much of the evidence remains indirect, correlational, or subject to methodological variability.

This framework posits that MDD emerges from an erosion of synaptic connectivity and glial support, leading to destabilization of large-scale brain networks.

Conversely, rapid-acting glutamatergic antidepressants exert their effects by unlocking synaptic plasticity and recalibrating maladaptive network states, producing rapid and sustained symptom relief [5,13,19].

Rapid activation of AMPA throughput, mTORC1, and BDNF–TrkB cascades, described in detail in Section 4, is hypothesized to initiate microcircuit plasticity. Here we focus on how these micro/meso-scale events scale to systems-level reorganization [14,35].

At the systems level, micro- and mesoscale plasticity manifests as a coordinated rebalancing across the three canonical networks, i.e., a resolution of the triple-network dysfunction described in Section 3: diminished dominance of the default mode network (DMN), strengthened engagement of the central executive network (CEN), and restored salience network (SN)-mediated switching between internally and externally oriented states. In this framework, the triple-network signature is not treated as a separate phenomenon but as the network-level readout of the molecular and cellular cascade outlined above; empirical details and effect sizes are reported in Section 7 [17,21]. While the Connectomic Glutamate Framework builds upon prior synaptic plasticity models and triple-network formulations, it differs in scope and structure. Traditional synaptic plasticity models emphasize molecular cascades (e.g., AMPA–mTOR–BDNF) without specifying how these changes propagate to whole-brain network dynamics. Triple-network models (DMN–CEN–SN) describe large-scale dysfunction but do not formally integrate molecular or glial mechanisms. These interpretations should be considered provisional, given the modest and heterogeneous effect sizes reported in neuroimaging studies and the limited reproducibility across cohorts.

Consistent with this account, the magnitude of connectivity normalization correlates with clinical improvement across studies, supporting a mechanistic link between plasticity-induced network reconfiguration and symptom change [73].

PET imaging with SV2A ligands provides a structural correlate, revealing that increases in synaptic density parallel network-level recovery, bridging microstructural plasticity with macroscale connectomic repair [29]. Nevertheless, the causal directionality between synaptic changes, network reorganization, and clinical outcomes remains unresolved. It is also important to note that the two-phase destabilization/reintegration sequence proposed in this review remains a heuristic abstraction. Current evidence does not demonstrate that ketamine, esketamine, or other glutamatergic agents uniformly follow this temporal structure, nor that the same plasticity mechanisms generalize across all compounds within the class.

### 8.2. Clinical Translation and Bidirectional Model

This mechanistic cascade can be conceptualized as a two-phase process [19,67]. The first is a destabilization phase, in which ketamine and related agents disrupt rigid network attractor states by producing a glutamate-driven perturbation of excitatory-inhibitory balance [35]. The second is a reintegration phase, during which synaptogenesis and glial support consolidate new circuit configurations, allowing networks to reassemble in more flexible, adaptive patterns [79,80,81]. In this sense, glutamatergic antidepressants can be viewed as “circuit re-tuners”, transiently perturbing pathological states and then stabilizing healthier dynamics through structural remodeling [79,82].

This two-phase framework is hypothetical, intended to integrate cellular and systems-level observations into a cohesive narrative [83]. Direct empirical evidence for this temporal sequence in humans is currently limited, as few studies combine longitudinal imaging, molecular biomarkers, and detailed phenotyping [84,85]. Areas requiring empirical validation include: (i) direct demonstration of mechanistic links between synaptic remodeling and network-level changes in humans; (ii) identification of patient-level moderators predicting adaptive vs. maladaptive network reintegration; (iii) longitudinal mapping of plasticity processes across days to weeks; (iv) integration of multimodal biomarkers (EEG, fMRI, PET, plasma/CSF markers); (v) experimental designs capable of testing causal relationships, such as pharmacological challenges, individualized stimulation paradigms, or computational modeling [86].

Clinically, this framework explains both the rapidity and durability of glutamatergic antidepressants. Immediate disruption of maladaptive network dynamics underlies rapid mood improvement, often within hours [12,87]. Individual variability across molecular or network domains may explain differences in treatment response and relapse risk [71]. This underscores the potential for precision psychiatry approaches, where multimodal biomarkers, fMRI, EEG, PET, guide personalized use of glutamatergic modulators [88].

**Figure 2:**
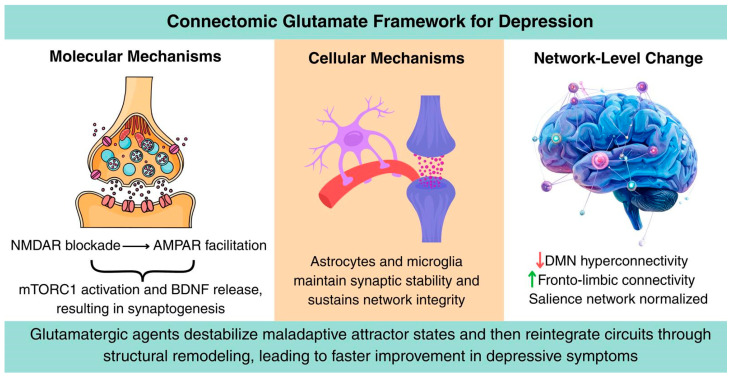
Summary of the Connectomic Glutamate Framework for Depression. NMDAR blockade increases AMPAR throughput, triggering mTORC1 activation and BDNF-dependent synaptogenesis. Astrocytes and microglia support structural remodeling and network stabilization, leading to reduced DMN hyperconnectivity, enhanced fronto-limbic integration and normalized salience-network dynamics.

In summary, the Connectomic Glutamate Framework proposes that MDD arises from synaptic erosion and network rigidity, and that rapid-acting glutamatergic antidepressants restore mental health through multi-level remodeling. This framework bridges molecules and connectomes, offering a unified explanation of depression and a roadmap for next-generation therapeutics (Figure 2).

### 8.3. Boundary Conditions and Patient-Level Moderators

Given the heterogeneity of MDD, the applicability of the Connectomic Glutamate Framework is likely to vary across depressive subtypes. Patients with prominent anhedonia, executive dysfunction, or elevated DMN hyperconnectivity may align more closely with the proposed mechanisms, whereas individuals with strong anxious-arousal features, trauma-related dysregulation, or comorbid psychotic symptoms may respond via partially distinct pathways. Likewise, baseline cognitive control, neuroinflammatory status, and genetic variables (e.g., BDNF Val66Met) may influence the extent to which glutamate-driven plasticity produces adaptive network changes. Future work is needed to delineate for whom—and under what neurobiological conditions—this framework provides the most accurate explanatory and predictive value.

## 9. Clinical Challenges and Research Priorities

The advent of rapid-acting glutamatergic antidepressants has reshaped the therapeutic landscape, yet key hurdles remain before widespread implementation. Within a connectomic framework, challenges cluster into three domains, including durability and safety, predictors and biomarkers, and implementation and equity.

### 9.1. Durability and Safety

A primary issue is durability, with a single ketamine infusion yielding marked improvement within hours, but benefits often waning within days to a week [11]. Maintenance schedules, requiring repeated infusions or intranasal esketamine, to extend efficacy, but the optimal balance of frequency, safety, and long-term tolerability is unresolved [12,61]. Real-world evidence suggests that durability may be lower outside clinical trials, with many patients requiring ongoing treatments at variable intervals. Data from community clinics and pragmatic trials indicate substantial heterogeneity in treatment trajectories, influenced by comorbidities, baseline severity, concomitant medications, and psychosocial context. From a connectomic perspective, this reflects the difficulty of stabilizing the acute “destabilization-reintegration” process into durable network reorganization. Consolidating rapid network resetting into stable remission is therefore a key translational priority.

Safety is a parallel concern. Ketamine produces dissociative and psychotomimetic effects, as well as transient elevations in blood pressure and heart rate; esketamine requires administration in certified centers with post-dose monitoring. Both agents raise concerns about misuse or dependency [89]. Although current data suggest that therapeutic use at controlled doses is safe, long-term outcomes remain insufficiently characterized. Chronic or repeated exposure raises additional questions about urological toxicity, cognitive effects and memory impairment, risk of misuse or dependence, Cardiovascular safety over months or years and potential neurotoxicity observed in animal models at high doses [89,90,91,92,93].

Long-term esketamine data are likewise limited, with existing extensions (e.g., SUSTAIN trials) spanning 1–2 years but lacking robust longitudinal neurocognitive or urological monitoring [94,95].

Overall, long-term tolerability remains one of the most pressing knowledge gaps and represents a key barrier to widespread adoption. These durability patterns challenge the idea that ketamine induces sustained network remodeling. The framework proposed here should therefore be interpreted as describing potential mechanisms of transient or partially stabilized plasticity, rather than a guaranteed pathway to long-term remission.

For dextromethorphan-bupropion, tolerability is generally favorable, yet vigilance is warranted regarding misuse at supratherapeutic doses [47]. Careful patient selection, clinical monitoring, and ongoing pharmacovigilance are therefore essential for safe deployment.

### 9.2. Predictors, Mechanisms, and Biomarkers

A second challenge is the heterogeneity of treatment response. Not all patients benefit from glutamatergic antidepressants, and robust predictors are still lacking. Clinical variables such as baseline severity, history of treatment resistance, and comorbid anxiety or substance use disorders may influence outcomes [87]. Neuroimaging has provided important leads, with patients with higher baseline DMN hyperconnectivity or lower prefrontal-limbic coupling being likely to respond [18,73]. Electrophysiological signatures, including increased gamma power and decreased frontal theta, also predict rapid improvement and could inform stratification [96]. Molecular factors, such as the BDNF Val66Met polymorphism, may further modulate efficacy [15]. The development of reliable multimodal biomarker panels remains a central research goal, with the potential to enable precision psychiatry approaches [97].

Mechanistic uncertainties compound this issue. The relative roles of R-ketamine, hydroxynorketamine metabolites, sigma-1 receptor signaling, and microglial modulation remain to be clarified. While preclinical and clinical data converge on the importance of synaptogenesis and glial support, strategies to enhance and stabilize these processes are still under exploration [16]. Future studies should prioritize longitudinal, multimodal designs integrating molecular biomarkers, neuroimaging, electrophysiology, and clinical outcomes, to validate mechanistic models and establish standardized biomarkers of glutamate-related network plasticity. Combination therapies, pairing ketamine with psychotherapeutic interventions, cognitive training, or neuromodulation, may help consolidate adaptive network states and reduce relapse risk [98]. Likewise, adjunctive agents that promote synaptogenesis or modulate inflammatory tone represent promising avenues for prolonging benefits. Understanding patient-level factors that predict adaptive versus maladaptive reintegration following network destabilization is a major unmet need and will be crucial to guide personalized treatment allocation.

### 9.3. Implementation, Access, and Equity

The third domain involves translation of glutamatergic treatments into practice, with equity of access emerging as a pressing issue. Current regulations restrict esketamine administration to certified centers with mandatory post-dose observation, limiting scalability [61]. Oral dextromethorphan-bupropion improves accessibility, but questions remain regarding long-term safety, drug–drug interactions, and cost-effectiveness [47]. Beyond regulatory constraints, substantial disparities exist worldwide, like the high treatment costs make ketamine and esketamine inaccessible in many low- and middle-income countries and the Lack of infusion centers or trained personnel limits availability even in high-income settings, particularly rural areas. Moreover, sociocultural and stigma-related factors may influence willingness to receive glutamatergic treatments, especially those involving dissociative experiences.

Even where treatment is available, systemic inequities may determine who receives rapid-acting antidepressants, exacerbating existing disparities in mental healthcare.

The use of ketamine or esketamine beyond TRD, for bipolar depression, suicidal crises, or in the adolescent population, remains under investigation [99,100].

High costs, infrastructure demands, and regulatory restrictions limit availability, creating disparities across healthcare systems and geographic regions [101]. Large-scale effectiveness studies, beyond highly selected trial populations, are required to assess real-world impact, health-economic implications, and integration into stepped-care frameworks. Addressing these challenges is essential to ensure that rapid-acting antidepressants benefit patients broadly, rather than only those in privileged care settings (Table 1 and Table 2).

## 10. Conclusions and Future Directions

MDD is now recognized as a disorder of impaired plasticity and maladaptive network dynamics, moving beyond the monoamine deficit model. Rapid-acting glutamatergic antidepressants have demonstrated that targeting excitatory transmission can induce rapid improvement, reshaping the therapeutic paradigm. However, the current evidence base remains limited by methodological constraints, modest sample sizes, heterogeneity across studies, and reliance on indirect biomarkers. Much of the mechanistic and connectomic literature is correlational, with causal relationships between synaptic remodeling, network reorganization, and clinical response still largely unproven. In this narrative review, we propose the Connectomic Glutamate Framework as a conceptual synthesis intended to integrate molecular, glial, and network-level findings into a cohesive, hypothesis-generating perspective.

The framework should not be interpreted as definitive or comprehensive; rather, it offers a heuristic that may help guide future research across multiple levels of analysis. Its scope is inherently constrained by gaps in the current literature and by the limited availability of longitudinal, multimodal human studies capable of directly testing its predictions.

Future priorities include enhancing durability, ensuring safety, and developing multimodal biomarkers to guide personalized treatment. Combining glutamatergic agents with pro-plasticity strategies or neuromodulation may stabilize adaptive states and reduce relapse.

The Connectomic Glutamate Framework provides a roadmap for translation, highlighting the challenge of embedding these insights into real-world care to deliver faster, safer, and more equitable treatments.

## Figures and Tables

**Table 1:** Glutamatergic agents in depression: preclinical mechanisms and clinical evidence.

Study (Year)	Design	Sample(s)	Key Findings	Clinical Implications
Berman et al. (2000) [11]	Randomized, placebo-controlled, crossover trial	7 depressed patients (MDD and bipolar)	Single IV ketamine (0.5 mg/kg) produced rapid antidepressant effects within hours.	First evidence of rapid antidepressant action of ketamine; opened the field for glutamatergic treatments.
Zarate et al. (2006) [12]	Randomized controlled trial	18 TRD patients	Single IV ketamine infusion produced robust and rapid antidepressant effects lasting up to 1 week.	Landmark trial demonstrates efficacy of ketamine in TRD; pivotal for clinical translation.
Li et al. (2010) [14]	Preclinical mechanistic study	Rodent models	Ketamine activated the mTOR pathway, increased synaptic proteins, and spine formation in the PFC.	Identified mTOR-dependent synaptogenesis as a mechanism for the rapid effects of ketamine.
Murrough et al. (2013) [102]	Two-site randomized controlled trial	73 TRD patients	Single IV ketamine produced a significant antidepressant response within 24 h compared to midazolam.	Confirmed rapid antidepressant efficacy in TRD; strengthened evidence base for ketamine.
Yang et al. (2015) [44]	Preclinical comparative study	Rodent models	R-ketamine produced more potent and longer-lasting antidepressant effects than S-ketamine without psychotomimetic side effects.	Suggested R-ketamine as a safer and more effective enantiomer for clinical development.
Miller et al. (2014) [103]	Review of mechanistic biomarkers	Not applicable	Discussed immune, inflammatory, and glutamatergic mechanisms underlying depression and ketamine response.	Highlighted translational biomarkers linking glutamatergic modulation to clinical efficacy.
Zanos et al. (2016) [45]	Preclinical mechanistic study	Rodent models	Identified (2R,6R)-hydroxynorketamine as an active ketamine metabolite with antidepressant-like effects independent of NMDA blockade.	Shifted focus toward ketamine metabolites as potential novel therapeutics.
Daly et al. (2019) [61]	Randomized, double-blind, relapse prevention trial	705 patients with TRD	Esketamine nasal spray, combined with oral antidepressant, significantly delayed the time to relapse compared to placebo and antidepressant.	Established the role of esketamine in maintenance treatment, led to FDA approval for TRD.
Yang et al. (2018) [71]	Randomized, double-blind, active-controlled trial	63 patients with TRD randomized to R-ketamine or S-ketamine	Evaluated effects of ketamine enantiomers on depressive symptoms; confirmed differential profiles.	Extended translational evidence for R-ketamine as a candidate treatment.
Iosifescu et al. (2022) [47]	Randomized, double-blind, controlled trial (GEMINI)	327 patients with MDD	AXS-05 (dextromethorphan-bupropion) demonstrated a significant improvement in MADRS scores compared to placebo.	Demonstrated efficacy and safety of oral NMDA/sigma-1 modulation in MDD.

FDA: Food and Drug Administration; IV: Intravenous; MADRS: Montgomery-Åsberg depression rating scale; MDD: Major depressive disorder; mTOR: Mammalian target of rapamycin; NMDA: N-methyl-D-aspartate; PFC: Prefrontal cortex; TRD: Treatment-resistant depression.

**Table 2:** Network-level studies in MDD and effects of glutamatergic modulation.

Study (Year)	Design	Sample(s)	Key Findings	Clinical Implications
Sheline et al. (2010) [2]	Resting-state fMRI	18 MDD, 17 controls	Identified “dorsal nexus” in dorsomedial PFC with abnormal hyperconnectivity across DMN, CCN, and AN. Connectivity strength correlated with depression severity.	Dorsal nexus may serve as a hub of pathological connectivity in MDD; potential biomarker and target for neuromodulation.
Hamilton et al. (2011) [30]	Resting-state fMRI	25 MDD, 25 controls	Elevated DMN activity linked to rumination; failure to deactivate DMN during tasks.	Rumination arises from the inability to disengage DMN; treatment should normalize DMN suppression.
Scheidegger et al. (2012) [17]	Double-blind, placebo-controlled, crossover fMRI	16 healthy subjects	Ketamine reduced connectivity between sgACC-mPFC and within DMN.	Provides first evidence of ketamine-induced network remodeling in humans.
Kaiser et al. (2015) [20]	Meta-analysis of rs-fMRI	27 studies (556 MDD, 518 controls)	Consistent hypoconnectivity within FPN and between FPN-DAN; hyperconnectivity within DMN and DMN-FPN.	Supports triple-network dysfunction; imbalance explains rumination and impaired cognitive control.
Abdallah et al. (2017) [73]	Resting-state fMRI (GBCr)	18 MDD, 25 controls	Reduced prefrontal global connectivity at baseline; ketamine normalized GBCr in PFC; responders showed increases in lateral PFC, caudate, and insula.	GBCr may be a biomarker of rapid antidepressant response; it supports the synaptic homeostasis model.
Gärtner et al. (2019) [18]	Prospective fMRI	24 TRD patients	Ketamine increased connectivity between the right lateral PFC and sgACC; low baseline FC predicted better response.	PFC-sgACC connectivity is a predictive and explanatory biomarker of ketamine response.
McMillan & Muthukumaraswamy (2020) [22]	Systematic review	33 ketamine neuroimaging studies	Ketamine generally preserves cortico-subcortical but disrupts corticocortical connectivity.	Highlights consistent ketamine modulation of networks; evidence for network-based biomarkers.
Zhou et al. (2020) [21]	Resting-state fMRI + graph theory	66 MDD, 62 controls	Increased connectivity in DMN and decreased integration in cognitive control networks.	Confirms DMN dominance and inefficient network communication in MDD.
Holmes et al. (2019) [29]	PET ([^11^C]UCB-J) + resting-state fMRI	26 unmedicated MDD/PTSD patients + 21 controls	Lower synaptic density (SV2A) in dlPFC, ACC, hippocampus correlated with depression severity and reduced functional connectivity between dlPFC and PCC.	First in vivo evidence linking synaptic loss to network dysfunction in MDD; supports synaptogenesis as a mechanistic target for rapid-acting antidepressants.
Scangos et al. (2021) [97]	Intracranial EEG + fMRI (precision psychiatry)	TRD patient, individualized	Identified state-dependent network biomarkers predicting mood fluctuations; guided targeted stimulation.	Illustrates the feasibility of personalized network-guided interventions in TRD.
Wade et al. (2022) [104]	Multimodal MRI + machine learning	60 TRD, 19 controls	Pretreatment anterior DMN-posterior insula connectivity and SLF microstructure predicted symptom reduction after serial ketamine.	Anterior DMN and insula connectivity may guide patient stratification; a potential predictive biomarker.

AN: Anterior network; CCN: Cognitive control network; DMN: Default mode network; EEG: Electroencephalogram; fMRI: functional magnetic resonance imaging: FPN: Frontoparietal network; MDD: Major depressive disorder; sgACC: Subgenual anterior cingulate cortex; TRD: Treatment-resistant depression.

## Data Availability

No new data were created or analyzed in this study.
